# Racial and Ethnic Representation in Dementia Clinical Trials Registered on ClinicalTrials.gov in the United States, United Kingdom, and Canada

**DOI:** 10.1002/gps.70211

**Published:** 2026-05-09

**Authors:** Daniel Kramarczyk, Clive Ballard, Anne Corbett, Miguel Vasconcelos Da Silva, Kamara Israel‐ Mcleish, Jeffrey Cummings, Zunera Khan

**Affiliations:** ^1^ Institute of Psychiatry Psychology and Neuroscience King's College London London UK; ^2^ University of Exeter Medical School University of Exeter Exeter UK; ^3^ University of Nevada Las Vegas Nevada USA

**Keywords:** Alzheimer's disease, clinical trials, dementia, ethnic populations, ethnicity

## Abstract

**Objective:**

To evaluate racial and ethnic representation and temporal trends in phase II–IV dementia clinical trials conducted in the United States, United Kingdom, and Canada.

**Methods:**

We interrogated ClinicalTrials.gov for interventional dementia and Alzheimer's disease (AD) trials completed since 2000. Data on age, gender, and ethnicity were extracted from 163 eligible trials. Representation was compared across two time periods (2000–2015 and 2016–2019) to assess progress in diversity.

**Results:**

Of the 163 trials, 58.9% (*n* = 96) reported ethnicity data. Among the 12,900 participant records in these trials, 80.6% were Caucasian. Since 2016, despite improved reporting standards (100% of recent trials reported ethnicity), actual diversity declined: Asian participant representation dropped from 4.9% to 1.2%, and Hispanic/Latino representation fell from 2.2% to 0.7%. No ethnic minority group showed an increase in participation over the study period.

**Conclusions:**

Diverse ethnic groups remain significantly underrepresented in dementia clinical trials, with diversity metrics stagnating or declining over the last decade. Greater inclusivity in trial design and recruitment is urgently required to ensure that emerging dementia treatments are safe and effective for all populations.

## Key Points

- Stagnant Diversity: Despite improved reporting of race and ethnicity in ClinicalTrials.gov, since 2016, there has been no corresponding increase in the inclusion of minoritised populations in dementia trials.
- Declining Representation: Participation from Asian and Hispanic/Latino communities has significantly decreased over the last decade, falling to approximately 1% of dementia trial populations.
- Clinical Generalizability: The overwhelming predominance of Caucasian participants (80.6%) limits the generalizability of new disease‐modifying therapies, necessitating culturally tailored outreach and inclusive recruitment strategies.

## 1 Background

Alzheimer's disease (AD) is a devastating neurodegenerative disorder characterised by progressive cognitive and functional decline, which is now a major public health concern [1]. There are approximately 6.7 million people with dementia in the US, and 50 million people worldwide, the majority of whom have AD or AD pathology, and prevalence is projected to increase to over 80 million by 2050 [2]. The development of better diagnostics and new therapeutics for the treatment and prevention of AD is, therefore, a top health priority. There have been significant developments in both the evolution of biomarker technologies to enhance the early diagnosis of AD and the emergence of a new class of disease‐targeted therapies that address amyloid pathology. Anti‐amyloid therapies for AD, lecanemab and donanemab, are now licenced for the treatment of AD in both the United States (US) and the United Kingdom (UK) [3, 4]. The licencing of these treatments has catalysed a significant increase in the number of new compounds moving forward in clinical development pipelines.

While this is a promising development for patients and the field, it is critical to ensure that trials of these emerging treatments have appropriate representation across ethnic communities to ensure they are safe and effective for all patients. There are significant differences in prevalence and contributing factors of AD across ethnic groups, with increased risk and prevalence in African American and Hispanic groups in the US, and in Black and Asian Minority Ethnic (BAME) communities in the UK [5]. This may in part be due to higher prevalence of key dementia risk comorbidities such as hypertension, cardiovascular disease, diabetes and stroke among South Asian and Black individuals compared to White Europeans. A major consideration for the new disease‐targeted treatments is the occurrence of amyloid‐related imaging abnormalities (ARIA), including vasogenic oedema and microhaemorrhages [6, 7]. The possible differential occurrence of ARIA across ethnic groups is unknown. Recent studies have also revealed distinct biomarker patterns, such as altered amyloid profiles in older individuals of Black heritage [8]. More recent studies reported that plasma amyloid–based screening eligibility differed across racial and ethnic groups in a preclinical AD trial screening cohort, although PET eligibility among those plasma‐eligible did not differ, highlighting how biomarker‐driven enrichment may influence who progresses through trial screening [9]. These issues may impact trial eligibility as well as treatment safety. Evidence from other therapeutic areas highlights differential treatment response between ethnic groups that may apply in the context of AD. For example, in the treatment of hypertension, there is a more favourable response to *β*‐blockers and ACE inhibitors in Caucasian populations than in people of African descent, while the reverse is seen with diuretics [10]. Similar issues apply to emerging treatments for neuropsychiatric symptoms such as agitation, aggression, and psychosis in people with AD. There are mixed findings regarding the troublesome adverse effects of atypical antipsychotics, which appear to be more pronounced in minority populations [11].

Recent analyses of ClinicalTrials.gov data have highlighted persistent limitations in the reporting and representation of racial and ethnic diversity in Alzheimer's disease clinical trials. In particular, Arellanes et al. (2024) [12] examined racial and ethnic representation in randomised Alzheimer's disease trials registered on ClinicalTrials.gov and demonstrated ongoing under‐representation of minoritised populations. The current study extends prior work by providing a more comprehensive and robust evaluation of racial and ethnic representation in dementia clinical trials. Specifically, we adopt a broader scope beyond Alzheimer's disease alone, incorporating trials of dementia more generally, including mild cognitive impairment and related comorbid dementia conditions, reflecting contemporary understanding of mixed pathology in dementia. In addition, our analysis is not restricted to randomised controlled trials but includes a wider range of interventional study designs, as well as terminated trials included as part of a sensitivity and robustness assessment. We further expand the geographic scope to include trials conducted in the United Kingdom and Canada, alongside the United States, and provide a more detailed breakdown of trial‐reported racial and ethnic categories. Importantly, we explicitly examine temporal trends in representation to assess whether diversity in dementia clinical trials has improved over time. Despite improvements in the reporting of race and ethnicity in more recent trials, our findings demonstrate no corresponding improvement in participant diversity and, for some groups, a decline. Given that the generalisation of trial results depends on enrolling populations that reflect those in whom treatments will be used, and in light of guidance from regulatory agencies including the EMA and MHRA emphasising the importance of inclusive trial recruitment [13, 14, 15, 16], as well as US Food and Drug Administration guidance recommending that sponsors broaden eligibility criteria and reduce unnecessary exclusions to improve representativeness of populations likely to use the therapy [9], the present study aims to report on racial and ethnic representation across dementia clinical trials in North America and the United Kingdom, and to inform recommendations for future trial design and delivery through the lens of inclusivity and representation.

## 2 Methods

### 2.1 Search Strategy

A search was undertaken for research trials registered on clinicaltrials.gov (15th September 2023). Inclusion criteria for the search strategy included condition/disease (AD), OR other terms (dementia), study type (intervention), location (UK, USA, Canada), study status (completed and terminated; active studies do not have demographic data reported on clinicaltrials.gov), phase (II‐IV) and study, trial results available. Detailed search elements, including specific terms and filters used in the ClinicalTrials.gov registry, are provided in Appendix A. This was agreed within the research team (CB, ZK, DK). Phase 1 studies are early‐stage trials that offer preliminary evidence, lacking the comprehensive data necessary for strong clinical conclusions. Therefore, these were not included in the current review aimed at examining ethnic representation in studies registered on clinicaltrials.gov. An additional search was undertaken on 19th June 2024 to examine the literature landscape between 15th September 2023 and June 2024, using the same criteria.

### 2.2 Data Extraction

Pairs of researchers independently screened study titles and overviews from CT.gov. The types that were included in the review focused on agreed‐upon search terms. For each trial, data were extracted for study name/number, condition, year of publication, study type, design, country and population demographics (age, gender and ethnicity).

Race and ethnicity were reported heterogeneously across trials and across regions. In United States–based studies, ethnicity typically referred to Hispanic or Latino origin, while race categories included White, Black or African American, Asian, and other classifications. In contrast, trials conducted in the United Kingdom and Canada more commonly reported combined ethnicity categories rather than distinguishing race and ethnicity as separate variables.

Trials were not excluded on the basis of how race or ethnicity was defined or reported, provided that demographic data were available in ClinicalTrials.gov.

The periods 2000–2015 and 2016–2019 in the sample cohort were chosen because of the observed difference in ethnicity data reporting practices. All trials that did not report race or ethnicity data were conducted between 2000 and 2015. While 62 trials within this period did report ethnicity, complete and consistent reporting was observed only in trials conducted from 2016 onwards. Therefore, grouping the data into these periods allowed us to highlight this marked change in reporting standards over time.

To maintain geographic comparability, studies were included only if recruitment sites were in the United States, the United Kingdom, or Canada. Multinational trials enrolling participants in additional countries were excluded to avoid combining demographic distributions from outside the target regions and to ensure the analysis reflected race/ethnicity reporting practices within these settings.

## 3 Results

### 3.1 Search Outcomes

**FIGURE 1:**
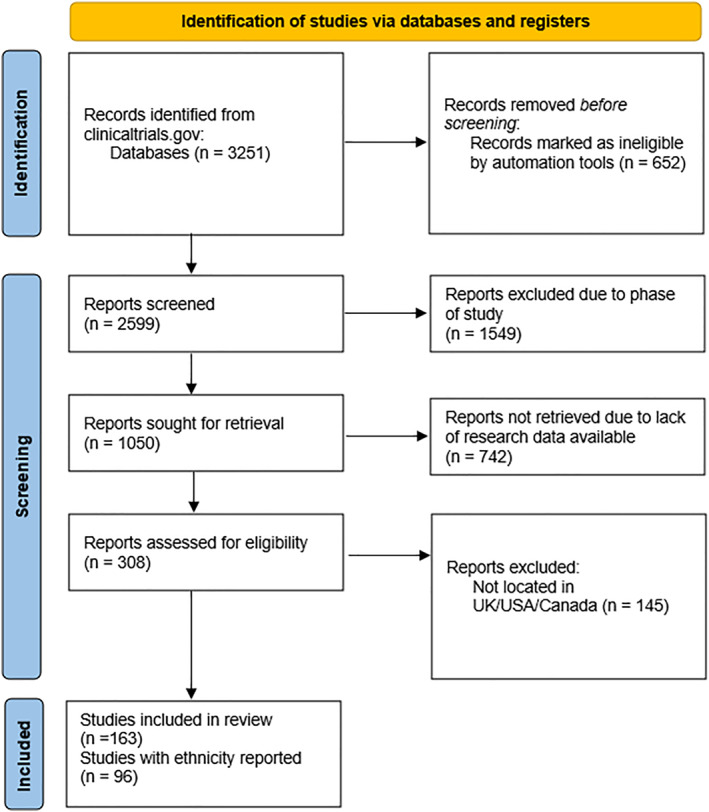
PRISMA flow diagram of the study selection and inclusion process. Trials were excluded if they included any sites outside the United States, United Kingdom, or Canada, even when sites within these countries were also present.

Initial data screening identified 3251 studies, of which 2599 were intervention‐based. Of these, 1050 were phase II‐IV studies, and 308 reported findings. From this group, 163 studies were included based on their focus within the UK, Canada, and the US. Within this subset, 96 studies reported on ethnicity, with 83 completed and 13 terminated. 145 studies were conducted in multiple sites around the world within countries from Europe, Asia and America. These were not included in the analysis (Figure 1). Some excluded multinational trials also included sites within the United States, United Kingdom, or Canada, but were excluded due to concurrent enrolment in regions that were not the focus of that paper. In a second search (19th June 2024), a further seven eligible studies were extracted. However, these studies were ongoing with no findings reported, so they were not included. 111 studies were randomised trials, 15 were non‐randomised, and 37 did not specify the design.

### 3.2 Participant Demographics

The included studies represented 24,480 participant records. Among the 163 clinical trials reviewed, 141 specifically targeted AD, while the remaining 22 trials focused on Mild Cognitive Impairment (MCI), unspecified dementia, and comorbid variations of AD, including dementia with Lewy Bodies (DLB), Parkinson's disease (PD), and vascular dementia (VD). Among the studies that reported age, 77% of participants were between 70 and 79 years old, making this the most common age range. 32 studies included participants under the age of 65. Gender was reported in all studies, with females accounting for 52% of the overall sample across the studies (Table 1). Due to the presence of five studies with sample sizes exceeding 1 SD above the mean study sample size, a sensitivity analysis was performed to ensure that these outliers did not skew the overall results (Table 2).

**TABLE 1:** Aggregated demographic characteristics of participants across included clinical trials.

Demographic	Category	%
Age	40–49	0.55
	50–59	2.31
	60–69	16.42
	70–79	77.32
	80 +	3.39
Sex	Female	52.16
	Male	47.84
Condition	Alzheimer's disease	86.5
	Mild cognitive impairment	6.13
	Dementia with lewy bodies/Parkinson's disease	2.45
	Vascular dementia	1.85
	Unspecified	3.07

*Note:* Percentages are calculated based on aggregated participant counts across all included trials reporting the respective variable.

**TABLE 2:** Sensitivity analysis comparing participant characteristics in trials below and above one standard deviation of the mean sample size.

	Below 1 SD	Above 1 SD
Ethnicity		
White	6198	4196
Non‐white	1533	973
White	39.55%	40.61%
Non‐white	10.45%	9.39%
Age		
Mean age (years)	71.61	72.83
Median age (years)	74.5	74.5
Sex		
Male participants	6166	4576
Female participants	6844	4703
Male participants	47.39%	49.32%
Female participants	52.61%	50.68%

*Note:* Trials were grouped by sample size. ‘Above 1 SD’ refers to trials with participant numbers greater than one standard deviation above the mean sample size across included studies; groups are mutually exclusive.

## 4 Ethnicity

Out of the 163 studies, 96 (58.9%) provided information on the ethnicity of the sample population, while 67 studies (41.1%) did not. Among the studies that reported on ethnicity, with 12,900 participant records, 15 were single race, predominantly featuring Caucasian groups. 81 studies included individuals from multiple ethnic groups. However, the distribution within these trials was predominantly Caucasian (80.6%) (Table 3).

**TABLE 3:** Aggregated ethnic background across participants in clinical trials reporting demographic data (*n* = 12,900).

Ethnic background across study populations in all ethnicity reported studies		*n* = 12,900
Ethnic group	Number of participants	Percentage
White	10,394	80.57%
Black or African American	775	6.10%
Asian	518	4.02%
Not Hispanic or Latino	784	6.08%
Hispanic or Latino	277	2.15%
American Indian or Alaska Native	21	0.16%
Native Hawaiian or Other Pacific Islander	9	0.07%
More than one race	50	0.39%
Unknown	72	0.56%

Of the 96 interventional trials that reported ethnicity data, 83 were conducted exclusively in the United States, with the remaining trials conducted in the United Kingdom and Canada. This distribution reflects the predominance of US‐based dementia trials within ClinicalTrials.gov and provides important context for the observed race and ethnicity reporting practices across studies.

### 4.1 Ethnic Diversity in Clinical Trials Over the Last Two Decades

The 67 studies that did not report ethnicity data were conducted between 2000 and 2015, whereas all 34 studies conducted post‐2016 did report ethnicity data. Between 2000–2015 and 2016–2019, there was a decrease in the proportion of trial participants of Asian heritage from 4.9% to 1.2%. Decreases were also seen in the Hispanic or Latino (2.2%–0.7%) group. There were minimal changes in other ethnic categories, including ‘Black or African American’, ‘American Indian or Alaska Native’, ‘Native Hawaiian or Other Pacific Islander’, and ‘Unknown’ (Table 4).

**TABLE 4:** Racial and ethnic representation in dementia clinical trials by time period.

Period	White	Black or African American	Asian	Hispanic or Latino	American Indian or Alaska Native	Native Hawaiian or Other Pacific Islander	More than one race	Unknown or other
2000–2015	82.85%	6.21%	4.86%	2.15%	0.16%	0.08%	0.48%	3.21%
2016–2019	81.27%	6.12%	1.24%	0.68%	0.20%	0.04%	0.08%	10.36%

*Note:* Percentages are calculated based on aggregated participant counts from trials reporting race and/or ethnicity within each time period.

## 5 Discussion

This study investigated the ethnic representation in dementia clinical trials conducted in the US, UK and Canada over 2 decades. The findings suggest that ethnic communities continue to be underrepresented in dementia clinical trials, indicating a pattern that has persisted and possibly even deteriorated over the last 2 decades. The implications of this lack of representation in trials are concerning and highlight the critical need to address health inequalities in AD interventional research.

Reporting of ethnicity in dementia clinical trials has improved substantially over the last decade. However, despite the improved reporting, there has been no improvement in the diversity of clinical trial populations. The representation of some communities, including Hispanic and Asian ethnicities, has reduced over the last decade. The current results, consistent with previous reports [16], demonstrate the ongoing significant underrepresentation of racial and ethnic minorities in dementia clinical trials. Despite extensive emphasis from regulatory bodies on the importance of including more diverse clinical trial populations, there has been no increase in representation over the last decade, and action is urgently needed to address this.

The most notable change identified in this review was the decrease in the representation of Asian populations in clinical trials conducted in North America and Europe over the last decade. This trend is particularly concerning given the rising prevalence of AD in South Asian communities, where the number of affected individuals is expected to double by 2026 [5]. Although this finding is limited to Asian participants in the US, Canada and the UK, the representation of Black and Hispanic participants has also remained notably low, with a slight decrease over the years. This overall decrease may reflect multifactorial barriers to participation in clinical research, such as language differences, healthcare access disparities, limited health literacy, mistrust of research, or lower recruitment efforts in these communities. Cultural and societal beliefs significantly influence research participation and preferences. However, limited research exists on this topic, highlighting the need for a deeper understanding of ways to improve research participation among underrepresented populations. Factors such as awareness, trust, logistical difficulties, and socioeconomic challenges may limit participation and engagement [17, 18]. Additionally, the stigma surrounding mental health often discourages individuals from discussing their health needs, creating another barrier to accessing services. To improve ethnic diversity in dementia trials, culturally tailored outreach, addressing barriers like cost and access, and setting diversity goals are essential. Building trust through community collaboration, transparent communication, and improved recruitment strategies, such as using inclusive and culturally sensitive language, could help connections with broader populations and foster research participation [19].

Issues related to differences in biomarker profiles between people from different ethnic backgrounds and the use of protocols that exclude participants with risk factors that are more prevalent in some non‐Caucasian populations [6] may also contribute to under‐representation. A review of protocol design will be important to avoid disproportionate exclusion of people from different ethnic groups due to these factors. Building such research infrastructure is vital to ensure that underrepresented populations are informed about dementia and research opportunities, while also helping researchers better understand participants' preferences through a holistic approach that acknowledges cultural and communication needs.

By extending prior registry‐based analyses to include a broader range of dementia diagnoses, interventional trial designs, geographic regions, terminated studies, and temporal comparisons, this study provides a more comprehensive assessment of racial and ethnic representation in dementia clinical trials.

This study included a large and comprehensive portfolio of trials conducted in the US, Canada and the UK. However, some limitations should be acknowledged. Interpretation of the findings was limited to some extent by the broad categorisation of different ethnic groups, and future studies would benefit from a more detailed categorisation of ethnic groups. For example, the specification of population subgroups would allow for the capture of within‐group variations. Such an approach could help to identify unique factors influencing participation in clinical trials in different ethnic groups. The study provides an overview of changes in ethnic representations over time, focusing on descriptive analysis of ethnic representation within trials registered on ClinicalTrials.gov. However, it does not offer any insights into the causality of the observations. A more detailed understanding of these factors will be crucial for developing effective strategies to address inequalities in research. Future research should investigate the underlying causes of underrepresentation. Potential factors such as mistrust, cultural stigma, logistical challenges, and socioeconomic disparities may hinder minority participation in dementia research.

Additionally, this study is limited by heterogeneity in race and ethnicity reporting across regions. In United States–based trials, race and Hispanic/Latino ethnicity are typically reported as distinct variables, whereas trials conducted in the United Kingdom and Canada more commonly use combined ethnicity categories. As a result, our use of the term “ethnicity” reflects trial‐reported classifications rather than a standardised demographic framework, limiting direct comparability across countries and highlighting the need for greater standardisation in demographic reporting in dementia clinical trials. Furthermore, the exclusion of multinational trials that included sites outside the United States, United Kingdom, and Canada may have resulted in the omission of larger global studies, which could differ in their recruitment strategies and representation of racial and ethnic minority populations.

This review underscores the urgent need for increased representation and more inclusive clinical trials. It emphasises the importance of improving engagement strategies and raising awareness about this issue across all communities. To achieve meaningful progress, tailored approaches are required to enhance engagement with diverse ethnic groups. Initiatives to address this clear inequality in AD trials are still in the early stages. However, there are growing research initiatives in dementia studies focused on improving participation [20, 21]. For example, the Community Ageing Research across Ethnicities (CARE) Network, established by King's College London, engages with diverse communities to gather input on unmet needs and receive feedback on research design, ensuring that research outputs are inclusive and culturally sensitive [22]. Initiatives like this will be critical to embedding change and true representation across clinical trial cohorts in the future.

## Conflicts of Interest

D.K. declares no known competing financial interests or personal relationships that could have appeared to influence the work reported in this paper. CB has received consulting fees from Acadia pharmaceutical company, AARP, Addex pharmaceutical company, Eli Lily, Enterin pharmaceutical company, GWPharm, H. Lundbeck pharmaceutical company, Novartis pharmaceutical company, Janssen Pharmaceuticals, Johnson and Johnson pharmaceuticals, Novo Nordisk pharmaceutical company, Orion Corp pharmaceutical company, Otsuka America Pharm Inc., Sunovion Pharm. Inc., Suven pharmaceutical company, Roche pharmaceutical company, Biogen pharmaceutical company, Synexus clinical research organization and tauX pharmaceutical company and research funding from Synexus clinical research organization, Roche pharmaceutical company, Novo Nordisk pharmaceutical company and Novartis pharmaceutical company. AC discloses financial relationships with Suven and Janssen pharmaceutical companies for consultancy work. Behrens, Alzheon, MedAvante‐Prophase, Acumen. MVD declares no known competing financial interests or personal relationships that could have appeared to influence the work reported in this paper. KIM declares no known competing financial interests or personal relationships that could have appeared to influence the work reported in this paper. JLC has provided consultation to Acadia, Acumen, ALZpath, Annovis, Aprinoia, Artery, Axsome, Biogen, Biohaven, BioXcel, Bristol‐Myers Squib, Eisai, Fosun, GAP Foundation, Green Valley, Hummingbird Diagnostics, Janssen, Karuna, Kinoxis, Lighthouse, Lilly, Lundbeck, LSP/eqt, Mangrove Therapeutics, Merck, MoCA Cognition, New Amsterdam, Novo Nordisk, Optoceutics, Otsuka, Oxford Brain Diagnostics, Praxis, Prothena, ReMYND, Roche, Scottish Brain Sciences, Signant Health, Simcere, sinaptica, T‐Neuro, TrueBinding, and Vaxxinity pharmaceutical, assessment, and investment companies. JLC owns the copyright of the Neuropsychiatric Inventory. JLC has stocks options in Annovis, Artery, Vaxxinity, ZK has received consulting fees from Acadia pharmaceutical company. This research is part funded by the National Institute for Health and Care Research (NIHR) HealthTech Research Centre in Brain Health. The views expressed are those of the author(s) and not necessarily those of the NIHR or the Department of Health and Social Care.

## Data Availability

The data that support the findings of this study are available on request from the corresponding author. The data are not publicly available due to privacy or ethical restrictions.

## Appendix A ClinicalTrials.gov Search Terms and Filters

**Table:**

Search element	Entry used
Registry	ClinicalTrials.gov
Search dates	15 September 2023 (primary search); 19 June 2024 (update search)
Condition/other terms (as entered)	Alzheimer disease OR dementia
Study type	Interventional
Phase	II–IV
Study status	Completed OR terminated
Results	Trial results available on ClinicalTrials.gov (results posted)
Location	United States OR United Kingdom OR Canada
Timeframe	Studies completed since 2000

*Note:* ‘Dementia clinical trials’ in this manuscript refers to the full eligible set identified using the above registry terms and filters, including Alzheimer's disease trials as the majority subset and related dementia presentations captured within ClinicalTrials.gov records.

## References

[1] P. Scheltens , B. De Strooper , M. Kivipelto , et al., “Alzheimer's disease,” Lancet 397, no. 10284 (2021): 1577–1590, 10.1016/s0140-6736(20)32205-4. 33667416 PMC8354300

[2] Alzheimer's Research UK , Dementia Statistics UK, (2022), https://www.dementiastatistics.org/statistics‐about‐dementia/prevalence‐2/.

[3] J. R. Sims , J. A. Zimmer , C. D. Evans , et al., “Donanemab in Early Symptomatic Alzheimer Disease: The TRAILBLAZER‐ALZ 2 Randomized Clinical Trial,” JAMA 330, no. 6 (2023): 512–527, 10.1001/jama.2023.13239. 37459141 PMC10352931

[4] C. H. van Dyck , C. J. Swanson , P. Aisen , et al., “Lecanemab in Early Alzheimer's Disease,” New England Journal of Medicine 388, no. 1 (2023): 9–21, 10.1056/nejmoa2212948. 36449413

[5] Alzheimer's Society UK. Black , Asian and Minority Ethnic Communities and Dementia Research, https://www.alzheimers.org.uk/for‐researchers/black‐asian‐andminority‐ethnic‐communities‐and‐dementia‐research2022.

[6] M. Patel , S. Abatcha , and O. Uthman , “Ethnic Differences Between South Asians and White Caucasians in Cardiovascular Disease‐Related Mortality in Developed Countries: A Systematic Literature Review,” Systematic Reviews 11, no. 1 (2022): 207, 10.1186/s13643-022-02079-z. 36176009 PMC9520891

[7] M. F. Hossain , A. U. Husna , and M. Kharel , “Use of Lecanemab for the Treatment of Alzheimer's Disease: A Systematic Review,” Brain Behav 14, no. 6 (2024): e3592, 10.1002/brb3.3592. 38867460 PMC11169267

[8] J. C. Morris , S. E. Schindler , L. M. McCue , et al., “Assessment of Racial Disparities in Biomarkers for Alzheimer Disease,” JAMA Neurology 76, no. 3 (2019): 264–273, 10.1001/jamaneurol.2018.4249. 30615028 PMC6439726

[9] D. P. Molina‐Henry , R. Raman , A. Liu , et al., “Racial and Ethnic Differences in Plasma Biomarker Eligibility for a Preclinical Alzheimer’s Disease Trial,” Alzheimer's & Dementia 17, no. 6 (April 2024): 3827–3838, 10.1002/alz.13803. PMC11180863 38629508

[10] J. A. Johnson , “Ethnic Differences in Cardiovascular Drug Response: Potential Contribution of Pharmacogenetics,” Circulation 118, no. 13 (2008): 1383–1393, 10.1161/circulationaha.107.704023. 18809808 PMC2730023

[11] S. Ormerod , S. E. McDowell , J. J. Coleman , and R. E. Ferner , “Ethnic Differences in the Risks of Adverse Reactions to Drugs Used in the Treatment of Psychoses and Depression: A Systematic Review and Meta‐Analysis,” Drug Safety 31, no. 7 (2008): 597–607, 10.2165/00002018-200831070-00005. 18558793

[12] I. Arellanes , L. Mirsafian , Y. Wang , and M. Jacobson , “Reporting and Representation of Participant Demographic Information in Completed Alzheimer’s Disease Clinical Trials,” Journal of the American Geriatrics Society 72, no. 9 (2024): 2868–2870, 10.1111/jgs.18958. 38748455

[13] Medicines and Healthcare Regulatory Authority , Increasing Diversity of People Taking Part in Research, https://www.hra.nhs.uk/planning‐and‐improvingresearch/best‐practice/increasing‐diversity‐people‐taking‐part‐research/2025.

[14] Core Clinical Sciences , EMA Trial Diversity Guidelines, https://www.coreclinicalsciences.com/news/ema‐trialdiversity#:~:text=The%20adopted%20guidelines%20include%20ICH%20E5%2C%20focusing%20on,E8%20%28R1%29%2C%20addressing%20General%20Considerations%20for%20Clinical%20Trials.

[15] European Medicines Agency , Patient Centricity and Inclusion in Clinical Trials, https://www.ema.europa.eu/en/documents/presentation/presentation‐patientcentricity‐inclusion‐and‐representativeness‐clinical‐trials‐ethical‐and‐academicperspective‐breakout‐session‐f_en.pdf.

[16] Fierce Biotech , Fda’s Diversity Guidance for Clinical Trials No Longer Available, https://www.fiercebiotech.com/biotech/fdas‐diversity‐guidance‐clinicaltrials‐no‐longer‐available2025.

[17] S. Franzen , J. E. Smith , E. van den Berg , et al., “Diversity in Alzheimer's Disease Drug Trials: The Importance of Eligibility Criteria,” Alzheimer's & Dementia 18, no. 4 (2022): 810–823, 10.1002/alz.064115. PMC8964823 34590409

[18] S. Pardhan , T. Sehmbi , R. Wijewickrama , H. Onumajuru , and M. P. Piyasena , “Barriers and Facilitators for Engaging Underrepresented Ethnic Minority Populations in Healthcare Research: An Umbrella Review,” International Journal for Equity in Health 24, no. 1 (2025): 70, 10.1186/s12939-025-02431-4. 40075407 PMC11905581

[19] A. Salman , C. Nguyen , Y. H. Lee , and T. Cooksey‐James , “A Review of Barriers to Minorities' Participation in Cancer Clinical Trials: Implications for Future Cancer Research,” Journal of Immigrant and Minority Health 18, no. 2 (2016): 447–453, 10.1007/s10903-015-0198-9. 25822567

[20] K. A. Ramirez , C. Gigliotti , E. A. Little , et al., “Overcoming Barriers to Latino Participation in Alzheimer's Disease Research,” International Journal of Aging and Human Development 100, no. 1 (2025): 23–40, 10.1177/00914150241268259. 39149977

[21] J. Cunningham‐Erves , Y. Joosten , S. V. Kusnoor , et al., “A Community‐Informed Recruitment Plan Template to Increase Recruitment of Racial and Ethnic Groups Historically Excluded and Underrepresented in Clinical Research,” Contemporary Clinical Trials 125 (2023): 107064, 10.1016/j.cct.2022.107064. 36572240 PMC9926351

[22] King's College London , Community Ageing Research Across Ethnicities ‐ CARE Network, https://www.kcl.ac.uk/research/community‐ageing‐research‐acrossethnicities‐care‐network‐1.

